# Farm Atmosphere: Calm Attention and Mobility Characterise Positive Horse Welfare

**DOI:** 10.3390/ani16101557

**Published:** 2026-05-20

**Authors:** Martine Hausberger, Noémie Lerch, Marine Grandgeorge

**Affiliations:** 1 Department Zoology and Entomology, Rhodes University, Makhanda 6140, South Africa; mhausberger.pro@gmail.com; 2 Centre National de la Recherche Scientifique, CEEC (Centre d’Etude en Éthologie et Cognition)—UMR 6552, University of Rennes, University of Normandie, F-35000 Rennes, France

**Keywords:** equid, time budgets, equid management, animal-based measures, attention, ethogram

## Abstract

On-farm welfare assessment of equines is a challenge given the large diversity of management practices, especially in terms of housing and activities. In our study, we tested time budget measures as a complementary tool to more conventional welfare indicators (e.g., stereotypic behaviours, ear position while foraging, neck shape). We observed 174 horses living in eight facilities (in their home environment) for which data on management practices and welfare were available. Time budget was assessed using the scan sampling method (1 min scan sampling over 30 min; 33 scans), while welfare assessment was based on classical measures. The study was performed in two parts: The first part consisted of validating time budget as a correlate of welfare state, which was performed at the individual level and according to the context of observation. In the second part, the data were analysed at the farm level by averaging data from all horses in the same facility. The results showed that the time spent feeding, in exploratory walking and observation behaviour were correlated with indicators of positive welfare and/or good practices in contrast to the time spent standing immobile resting or in fixed attention and/or in negative social interactions. Time budget varied greatly between facilities, reflecting welfare state and management (feeding, working) practices. This study shows that, provided that observations are made at different time periods when animals are calm, three sessions of 10 min. could give a good account of the local «atmosphere». These findings bring new insights into both methodological approaches and the significance of behaviours and, in particular, the importance of precisely defining and measuring the types of attention as a window into an animal’s internal state.

## Simple Summary

Assessing the welfare of horses on the farm level is particularly complex because their living conditions, such as housing and activities, can vary greatly. In this study, we tested whether observing how horses spend their time—known as a time budget—could help evaluate their welfare. We observed 174 horses across eight different facilities, focusing on their daily activities like feeding, walking, resting, or interacting with others. We compared these behaviours to welfare indicators (e.g., stereotypic behaviours, ear position while foraging, neck shape). The results showed that horses who spent more time in exploratory walking or observation behaviour had a better welfare rating. In contrast, horses who spent more time standing resting or in fixed attention, or engaging in negative social behaviours, tended to show more indicators of poor welfare. These results suggest that measuring how horses spend their time could be a useful and practical way to assess their well-being on a daily basis.

## 1. Introduction

Behaviour is the best window into how horses perceive their current situations: any, even slight, discomfort or pain induces behavioural or postural changes that, if identified, are a valuable source of information on the animals’ subjective state [1,2]. Because welfare is a subjective state [3], animal-based measures are the first step in welfare assessment in facilities. If behavioural problems are detected, then a thorough examination of the management practices may help identify the source of the problem. Most current protocols of welfare assessment are a combination of animal-based and resource-based measures. Such a mixed approach, which, in the case of on-farm assessments, needs to be rapid and non-intrusive, leads to a reduction in the part of the assessment devoted to animal-based, especially behavioural, measures. There is therefore a risk of under-evaluation of horse welfare per se (e.g., [1]). On-farm welfare assessment is indeed a challenge given the variety of management practices in horse facilities in terms of housing, feeding and working conditions (e.g., [4]). The applicability of the same protocols in different contexts (e.g., single-stall versus group outdoor housing) is therefore under question (e.g., [5,6,7]). Moreover, the lack of measures indicative of positive welfare, i.e., when horses are satiated and content (e.g., [6,8,9]) and their basic needs are fulfilled [10], is a problem that has led to using human qualitative assessments that may not be objective enough (e.g., Qualitative Behavioural Assessment or QBA [1]). In quiet contexts (without breeding or predators) with unlimited resources, adult feral or semi-feral horses spend time grazing, resting (lying or standing), observing and in slow locomotion, while more «active activities», such as trot, canter, and social interactions, occupy little time (e.g., [11,12,13]). These convergent observations may give a picture of what a «content» horse looks like, especially because contentment, as a low-intensity positive emotional state, fulfils the criterion of «calmness» that may be at the core of positive welfare [14].

Recently, Auer and colleagues [15] proposed that time budget could be a useful tool for assessing welfare, which, in view of the above-mentioned findings, indeed sounds quite promising. However, these authors also mentioned that comparisons between studies assessing time budgets were barely possible given the high diversity of methods and definitions of activities used, preventing any meta-analysis from being done. There are, nevertheless, indications that horses’ time budgets are influenced by environmental conditions, such as, for example, in winter conditions, where an increase in standing has been observed compared to summer, as a way of reducing energy expenditure [16]. Time budget reflects resource availability (e.g., reduced feeding in the absence of roughage, e.g., [17]) but also the internal state of the animal, which may, for example, reduce feeding activity when in pain (e.g., [18]). In these two last examples, the situation is problematic and requires that measures for remediation be provided; hence, the measure of time budget provides useful information on horse welfare. Some normal behaviours, such as alarm (vigilance) postures that occur when a horse is confronted with a fear-inducing stimulus, may become a sign of compromised welfare if they occur frequently (e.g., [19,20]). Thus, whereas the presence of such a behaviour is not a problem nor a valid measure per se, if horses spend a significant part of their time budget in this posture, it may be indicative of a more permanent chronic anxious state. What remains to be done in order for this behaviour, like some others, to constitute a sign of “abnormality” (i.e., here, of compromised welfare) is to establish the threshold of “normality” (i.e., uncompromised welfare). There is therefore a need for further comparable studies on a variety of situations. When considering on-farm welfare assessment, the limitations of time budget assessments are that they require very precise definitions of the behaviours observed. Although good ethograms have been published and widely applied (e.g., [21]), not all studies use them, or not in the same way, and sometimes, there is no list of the behaviours observed [15].

In the present study, we hypothesised that measures of time budget at the farm level would give an overview of the «behavioural atmosphere» in each facility and would be especially appropriate for identifying representations of contentment and positive welfare. However, because of the diversity of management practices between facilities, the context of observation (e.g., in-stall versus on-pasture) of time budget may be a limiting factor for comparisons. Nevertheless, here, we looked for characteristics that could be validated across contexts so as to design a protocol adapted to most farms. Therefore, this study has been divided into two parts. Part 1 consisted of assessing the relationships between time budget, welfare indicators and management practices at the individual level and within one given context of observation (i.e., pasture/paddock or indoor stall). Part 2 was devoted, using the same measures, to making assessments and comparisons at the farm level using the individuals’ averaged time budgets and welfare/management measures.

## 2. Materials and Methods

For more details, see Grandgeorge and collaborators [22].

For this study, and in order to ensure that we would find horses in positive welfare (i.e., good conditions), we took advantage of data from an earlier study [22] on welfare states and management practices in facilities, some of which offered group outdoor housing, roughage ad libitum, and low-intensity working activity [23]. Recent studies have suggested that some differences between facilities in terms of welfare and the human–horse relationship may be at least partially explained by working conditions [24,25], which were therefore taken into account here, like other management factors. Thus, we compared data on time budget measured in the home environment to those obtained (and published) earlier from the same facilities and horses on living and working conditions, as well as on welfare indicators [22].

Moreover, since we aimed to develop an on-farm welfare assessment, we tested whether a rapid assessment of time budget per horse (3 sessions of 10 min per horse) could generate data of interest for welfare assessment. Finally, we also tried to develop and propose more precise descriptions of some activities (e.g., activities when the horse’s body is immobile; type of walk) than those provided in the classical equine ethograms.

### 2.1. Ethical Statement

The experiments were carried out in 2019–2020 in accordance with Directive 2010/63/UE of the European Parliament and the Council on the protection of animals used for scientific purposes. They complied with the current French law related to animal experimentation (decree n° 2013 ± 118 of February 2013) and its five implementation orders, JO 7 February 2013, integrated into the Rural Code and the Code of maritime fishing under n° R.214 ± 87 to n° R.214 − 137. The experiments performed in this study were not within the scope of applying the European directive; thus, in accordance with this directive and the current French and Irish laws, the experiments described below did not require us to request authorisation. Ethical review and approval were waived for this study because (a) the experiments involved only behavioural observations and non-invasive interactions with the horses and (b) the horses used in this research were not research animals. Animal husbandry and care were under the management of the riding school staff. The riding school managers gave the authors verbal informed consent for this study, and verbal informed consent was obtained from the owner of the animals involved in this study (same as [22]).

### 2.2. Subjects, Facilities and Management Practices

Observations were made in 2019 and 2020 according to the facilities. One Irish and 7 French (located in a variety of regions) equestrian facilities were included in this study. All these facilities were riding schools that also offered equine-assisted interventions.

A total of 174 equids were included in this study: 77 mares and 97 geldings, aged 4 to 27 years (mean ± SE = 14.1 ± 0.4 yo), from various breeds but mostly ONC (French abbreviation for horses that do not belong to one of the official breeds recognised by the French registration service), which can be crossbreds or from unknown origin. Both ponies and horses were involved (FEI categorisation: pony ≤ 1.48 m at withers or horse > 1.48 m) (all named horses in the present paper for ease of reading). All subjects had been in the facility for at least one year.

Welfare assessment and observations of time budget were performed in parallel during the same time period for each facility and by the same observer (NL), who, therefore, was not blinded (but had not analysed one or the other at the time of observations and thus had no precise idea of what the results would be for each aspect).

The management in the 8 facilities was representative of the diversity of practices in equestrian professional systems, although none kept horses permanently in single stalls (Table 1). The duration of daily time turnout varied largely between sites, from 30 min to 24 h per day (mean ± SE = 7.2 ± 0.7 h). When outdoors, all equids were in stable groups (i.e., same group of horses together every day for at least several months at the time of study). Seventy four equids were kept permanently outdoors in collective pastures (i.e., grass present; 57 equids; group size between 2 and 34 equids) or paddocks (i.e., bare ground; 17 equids; one group of 7 and one group of 10 equids), 47 were housed indoors in collective stalls with daily time (>3 h/day) in pasture (29 equids; groups between 4 and 34 equids) or in paddock (18 equids; groups between 2 and 4 equids) and 53 equids were housed indoors in individual stalls with daily time on pasture (32 equids) or in paddock (21 equids).

All equids had access to water ad libitum and had hay, but not all horses received additional pellet or cereal meals. Those kept on pastures had access to grass. For data analyses, two categories were considered for hay: ad libitum access (130 equids) or restricted access (2 kg to 6 kg per day; 44 equids; 33 in facility 8 and 1 or 2 in each other facility). The number of meals of commercial pellets per day was also noted (no meal: 94 equids; 1 meal per day: 8 equids; 2 meals per day: 48 equids; or 3 meals per day: 24 equids).

The time spent working per week was recorded for each equid, and two categories were made according to the median number of hours worked per week ([0–5.5 h] and [5.5–14 h] per week). Concerning the working modalities, 19 equids had never been ridden, and 34 never did groundwork. One hundred and thirteen equids worked with a bit, and 51 never wore a bit.

### 2.3. Welfare Assessment (See Also [22] and Figure 1)

Welfare assessment had been performed from animal-based measures, i.e., welfare indicators [14], for all facilities, using health (body lesions, body condition score, neck shape), postural (ear positions while foraging), and behavioural (stereotypic and abnormal behaviours, reactions to humans in approach–contact and saddle tests) [22]. Since most welfare assessment protocols are defined for animals housed indoors, we chose to apply or adapt indicators that could be used for equids kept both in stalls and outdoors.

**Figure 1:**
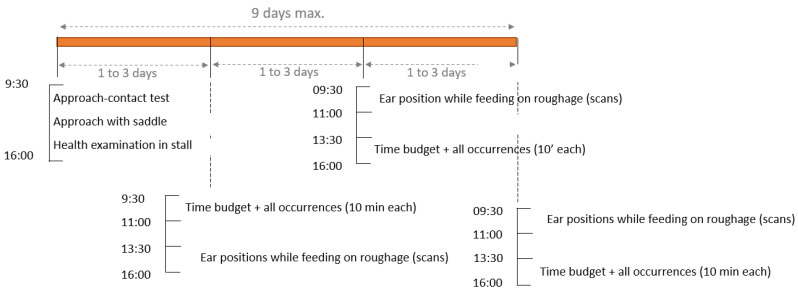
Observation protocol in each facility for welfare and time budget assessments, describing the time slots in which the different tests (human–horse relationship tests) and observation sessions took place. Descriptions of the activities recorded during time budget sessions and behaviours during all occurrences can be found in the Section 2.

Some indicators of compromised welfare, such as “depressed” states [26,27] or facing a wall [28], were not observed in the study population, even for those housed mostly in single stalls.

The measures and rationale for the choice of indicators are explained in Grandgeorge et al. [22], but the following indicators were used:

- Health indicators (observed once on a horse in its daily environment): The presence or absence of body lesions (hairless patches, scabs, skin lesions, wounds and marks of former wounds (white hair areas) related to the working equipment); body condition scored in five points (from 0 = emaciated to 5 = obese; subjects were divided into two categories: “optimal” (3–3.5) or “overweight” (4–5); neck shape as an indicator of spine state (flat or hollow necklines being associated with more tension along spine, hence back problems [29,30] and more welfare problems overall [31,32,33]. We followed Lesimple et al.’s [30] protocol for assessing neck shape.
- Postural indicator through ear positions: The time spent with ears backwards while feeding on roughage or grass has been correlated with chronic welfare impairment, such as vertebral disorders or stereotypic behaviours [23,34]. For each subject, 15 ear positions were recorded during different scan sampling sessions lasting 30 min (one scan every 5 min), when the equid was feeding on roughage (hay, straw or grass). The scan sampling session ended before the 30 min mark if the animal stopped feeding. During the observation, the experimenter walked slowly and regularly (1 step/second), 2 m away from the stalls in stables or from the edge of the field. Only when the equid continued feeding and paid no attention to the experimenter, the instantaneous ear position of the feeding equid was silently noted. The observer then resumed her walk up to the next subject.
- Behavioural measures in the home environment:
  (a) Stereotypic/abnormal repetitive behaviours (SB/ARB, pooled as “SB”) correspond to repetitive (repeated at least three times in succession and observed at least 5 times) behavioural sequences, performed with no obvious goal or function and are considered to reflect chronic stress [35]. The number of SB and ARB was recorded for each equid during three sessions lasting 10 min, following an “all-occurrences” sampling protocol [36]. The same SB and ARB as in Lesimple and collaborators [37] were recorded.
  (b) Acoustic signals (snorts) were recorded as possible indicators of positive emotions [38].
- Reactions to the experimenter in two horse–human relationship tests:
  (a) Approach–contact test: After entering the box, the experimenter waited for the horse to resume its earlier activity (e.g., eating) and positioned herself perpendicular to the horse’s shoulder. She then started, at a distance of 1.5 m from the horse, to move towards it at a speed of one step per second (i.e., 2 steps to touch the horse), with her arms at her side until she reached the animal’s shoulder, which she then tried to touch with her hand. The test stopped when the experimenter touched the horse. A case where the horse moved away before she had touched it was considered a failure, and the test was repeated (for a max. of 3 trials). The procedure was carried out on each side of the horse, starting on the left or right, in a random order between horses. Observations were obtained using continuous focal sampling, and the total number of positive or negative behaviours directed towards the experimenter was noted.
  (b) Saddle test: During the saddle test, the experimenter suddenly appeared at the door/entrance while the animal was feeding on the ground and opened it so that the equid could see the saddle. The first reaction (= change of behaviour) of the horse was noted as negative (ears backwards while gazing—score D—and/or approaching—score E—the experimenter), positive (ears forward while gazing—score B—and/or approaching—score A—the experimenter) or absent (score C: no reaction = no change of behaviour). In the present study, the scores were grouped according to their valence (A + B versus D + E versus C). The data were analysed in terms of one–zero at the individual level and the proportion of horses with each type of reaction at the facility level.

For these two tests, the animals were isolated (but in view of their group members or neighbours) and tested in a familiar environment (stall or part of a pasture).

### 2.4. Time Budget

Time budget was assessed using a scan sampling method [36] in which the animal’s activity was recorded at each scan. The list of activities and their definitions is indicated in Table 2. Whereas most activities are classically described in horse ethograms (e.g., [21], Supplementary Table S1), we put special emphasis on the locomotory and attentional components. Thus, we distinguished an exploratory walk (reflecting a relaxed type of locomotion) and an active walk, observed in more tensed situations, such as in paddocks with a high social density and low roughage availability [17,39]. We also distinguished two types of visual attention. Indeed, earlier studies on different species led us to distinguish different types of visual attention that may reflect species-specific characteristics (e.g., dogs use a more fixed attention, i.e., gazing, when looking at humans, while cats use a more mobile attention, i.e., glances when looking at humans [40]), but also the internal state of the animal (e.g., [41,42,43]). In horses, observation behaviour (i.e., mobile scanning of the environment) has been correlated with better health and welfare states [25,44], whereas fixed attention (gazes) has been more frequently observed in compromised welfare states [25]. The two types of spontaneous visual attention have also been observed in attention tests, and their respective use was influenced by horse working activity [45].

Three sessions lasting 10 min, with one scan per minute (total = 33 scans), and on different days (Figure 1), were performed for each individual during calm periods (i.e., outside meal time and working activities) in the animal’s main home environment (stall or pasture; Table 1) in the morning (9.00–11.30 a.m.), mid-day (11.30 a.m.–2.00 p.m.), and afternoon (2.00–4.30 p.m.). Sessions were made opportunistically (so as not to interfere with the facility’s routine), and the order of sessions (e.g., morning–afternoon) was semi-random. Data are expressed in % of “time” (= % of scans) spent by the horse in each activity.

### 2.5. Data Analysis

Data analysis differed between the two parts of the study.

- Part 1.

The data were analysed at the individual level and separately for each time budget observation context (outdoors: N = 114, horses from facilities 1 to 6; indoors: N = 60, horses from facilities 7 and 8). Each of the actual management (e.g., time spent outdoors) and welfare measures (number of stereotypic behaviours, number of snorts) was used for each individual; this method was also applied for individual time budgets (i.e., individual time spent in each activity, in % of scans).

For some management factors, the manager gave us a range of values. Thus, the horses were distributed into 4 categories based on the number of hours worked per week (0–3, 3–5.5, 5.5–11, 11–14 h) and 3 categories based on the number of concentrate meals: 0, 2 or 3 per day. For other management factors, the horses were divided based on a yes/no classification: roughage (ad libitum or restricted), ridden/not ridden, work bitted/bitless, and groundwork/no groundwork. If the horses could be used for both “ridden” and “groundwork”, they would then be registered in both or only one of these categories.

Concerning welfare measures, some were also considered in a categorical way: presence/absence of stereotypic behaviours, round/non round (i.e., flat or hollow) neck, more/less than 50% time spent with ears forward and with ears backwards while eating roughage on the ground, and presence/absence of positive and of negative behaviours during each of the human–horse relationship tests.

- Part 2.

Because our level of analysis was the facility level, we averaged the data for all horses in each facility that we compared with the prevalence (in % of horses) values linked to a management (e.g., indoor housing) or working (e.g., groundwork) factor. For the management factors, categories were established. When the parameters were continuous, the median was calculated (e.g., number of working hours per week and number of hours outdoors) to obtain the most balanced categories. For the other parameters, the results were in % of horses with this characteristic per facility. Thus, the categories studied were the % of horses that were housed 24 h outdoors or less than 24 h outdoors, had ad libitum or restricted foraging possibilities (roughage: grass or hay), had at least one meal of pellets or no meal of pellets, spent more than 5.5 h working per week or less than 5.5 h working per week, and were used for ridden work or not, used for groundwork or not, and used for work with a bit or without bit.

Time budget was analysed as the mean of the individual time budgets (% of scans) per facility. Thus, we calculated the mean time spent in each activity by each facility’s horses.

### 2.6. Statistical Analyses

All statistical analyses were performed using R software (version 4.0.2) created by R Core Team in 2018. The analysis was performed separately for the time budget, management, and welfare parameters.

- Part 1.

For analyses of non-categorical measures at the individual level, correlations were performed between time budget (% of scans for each activity) and the number of behaviours observed in the all-occurrences sessions (e.g., number of stereotypic behaviours) or the time spent in a given posture (e.g., % scans with ears forward) for welfare data or the time spent outdoors for management factors. For categorical measures, comparisons of time budgets were made (e.g., horses showing positive reactions in the approach–contact test versus horses that did not show any such reaction) using Mann–Whitney U tests.

- Part 2.

Analyses at the facility level were conducted using the catdes function (FactoMineR package) [49] and data for each individual, where each equestrian facility was characterised according to the significantly more represented management factors by calculating a test value [50]. The v-test facilitates a description of the classes of a partition (e.g., after an automatic classification). Test values are calculated for each continuous variable or category of a qualitative variable. They are measurements of the distance between the within-class value and the overall value. Correlations between time budget and management were calculated at the facility level, using the mean time budget per facility, on the one hand, and the proportions of individuals in each management type, on the other hand. An exception was made for the time spent outdoors because the horses in the category “less than 24 h outdoors” included horses that spent 21 h outdoors and others that spent 30 min outdoors; therefore, the median time outdoors per facility was used, not the proportion of horses.

## 3. Results

Part 1: Time budget, welfare state and management factors at the individual level and according to the context of observation.

All data are shown as a percentage of the 33 scans (e.g., 70.99% of the 33 scans were spent foraging). Horses spent most of their time feeding (foraging/grazing) (X = 70.99 ± 20.03) and resting (standing) (X = 14.45 ± 15.36). In the remaining time, activities were distributed between visual attention (fixed attention: 5.6 ± 7.52; observation: 1.78 ± 2.37), locomotion (exploratory walk: 0.63 ± 1.07; active walk: 0.52 ± 0.88), maintenance (0.78 ± 1.24), and social behaviours (positive: 0.40 ± 0.72; negative: 0.49 ± 0.86). No recumbency, play behaviours or higher locomotion (trot, canter) were observed.

### 3.1. Time Budget and Welfare Measures

In each context of observation, there were a number of correlations between time budget and welfare measures, with some activities associated with rather positive welfare measures and others with indicators of compromised welfare. All significant correlations are presented in Table 3.

- Time budget observations in stalls.
  Activities that were related to positive welfare indicators:
  - Feeding: The more time the horses spent feeding, the more time they spent with their ears forward while foraging and the less time they spent in stereotypic behaviours and with their ears backwards while foraging. The horses that had positive welfare indicators spent more time feeding, such as those that expressed positive reactions in the saddle test and those that spent more than 50% of their time with their ears forward during foraging.
  - Positive social interactions: The time spent in positive social interactions was higher in the horses that had a round neck shape (i.e., better back health).
  - Observation behaviours: The time spent in observation tended to be lower in the horses that reacted negatively to the approach–contact test.
  - Exploratory walk: the time spent in exploratory walk tended to be higher in the horses that had positive reactions in the saddle test.

Activities that were related to negative welfare indicators:

- Fixed attention (gazing): The time spent gazing was positively correlated with the time spent with ears backwards while foraging. Moreover, the horses that had negative welfare indicators spent more time gazing, including the horses that expressed stereotypic behaviours and those that spent most time with their ears backwards while foraging.
- Resting standing: The more time the horses spent resting standing, the more time they spent with their ears backwards and the less time they spent with their ears forward while foraging. Moreover, the horses with positive reactions in the saddle test spent less time resting standing.

- Time budget observations outdoors.
  Activities that were related to positive welfare indicators.
  - Feeding: The horses that expressed positive reactions in the saddle test tended to spend more time feeding
  - Exploratory walk: The time spent in exploratory walk was higher in the horses that spent more than 50% of their time with their ears forward while foraging.
  - Positive social interactions: The time spent in positive social interactions was higher in the horses that had a round neck shape (i.e., better back health); these horses also spent more time in maintenance behaviours.
  - Observation behaviours: Observation behaviours were noted less often in the horses that reacted negatively to the saddle test.
  Activities that were related to negative welfare indicators.
  - Resting standing: The more time the horses spent resting standing, the more time they spent with their ears backwards and the less time they spent with their ears forward while foraging.

To summarise, despite the differences related to the context of the time budget observations, some activities clearly stood out as correlates of a better welfare state in both situations: feeding (especially in the indoor situation and in relation to a better human–horse relationship), exploratory walk (especially in the outdoor situation), and social positive interactions that were observed more in the horses with a round neck, while observation behaviours were observed less in the horses that had a negative human–horse relationship. This was confirmed by the fact that the horses that spent more time feeding, observing and being in positive social interactions produced more snorts (outdoors). On the contrary, immobility, such as resting standing (both situations) and fixed attention (indoors), was more related to negative welfare.

Finally, it is interesting to note that the number of snorts (as an acoustic marker of positive emotions, mostly heard in horses housed outdoors) recorded during the all-occurrences sessions of the welfare assessment was positively correlated with three activities in the time budget, i.e., feeding, observation behaviours, and positive social behaviours, and negatively with the time spent in negative social interactions.

### 3.2. Time Budget and Management Conditions

- Time budget observations in stalls.
  Activities that were related to positive welfare indicators:
  No such significant correlation was observed (all *p* > 0.05).
  Activities that were related to negative welfare indicators:
  - Feeding: The horses that had restricted roughage availability, two or three meals of concentrates, and worked bitted spent less time feeding than the other horses.
  - Active walk: The time spent in active walk was higher in the horses that had restricted access to roughage.
  - Fixed attention: The time spent gazing was higher in the horses that received three or more concentrate meals and in the horses that worked with a bit.
  - Resting standing: Resting standing was observed more in the horses that worked with a bit.
  - Negative social interactions: The horses spent more time in negative interactions if they had three or more concentrate meals.
- Time budget observations outdoors.
  Activities that were related to positive welfare indicators:
  - Feeding: The horses that had roughage ad libitum and were not ridden spent more time feeding. Observation behaviours: The horses that had roughage ad libitum spent more time in observation behaviour. Resting standing: The horses that had three or more concentrate meals spent more resting standing, which was also the case for those that worked more than 3 h per week. Active walk: The horses that were never used for groundwork spent more time in active walk. Fixed attention: The horses that received more concentrate meals spent more time gazing and those that were ridden with a bit.

Activities that were related to negative welfare indicators:

No such significant correlation was observed (all *p* > 0.05).

To summarise, correlations appeared between the same main management factors (feeding practices, working practices) and time budgets, despite the differences in the context of observation. A higher ratio of concentrates/roughage was associated with horses spending more time in fixed attention (both contexts), resting standing (outdoors) and active walk (indoors) and less time feeding (both contexts), while roughage ad libitum was associated with more time feeding and in observation behaviour in the horses observed outdoors. Working with a bit was associated with more time in fixed attention in both contexts of observation, more time resting standing (indoors) and less time feeding (indoors), while the horses observed outdoors that worked more than 3 h per week also spent more time resting standing.

Part 2: Time budget as a tool for assessing welfare and good management practices in on-farm assessments.

In order to test whether the short assessment of time budget could be applicable for on-farm welfare assessment, and given the results of Part 1, we pooled the different facilities independently of the context of observation.

### 3.3. Facility Management and Welfare Profiles

See also Grandgeorge and collaborators [22] for more details.

The facilities visited clearly had different management practices, as shown by the results of the v-test (Table 4). Thus, although no facility kept its horses permanently indoors, three facilities (2, 7, 8) allowed less time in free outdoor turnout to their horses than the five other facilities. Facility 8 gave more concentrates, and fewer horses were given ad libitum access to roughage. The facilities also differed greatly in terms of working conditions, whether in terms of quantity, with less time working (in terms of hours per week) in facilities 2, 7, and 8, or in terms of type of activity, with more horses doing groundwork in facilities 5, 7 and 8 and more ridden work in facility 3. More horses were bitted for work in facilities 4, 5 and 6 and fewer in facilities 1, 2 and 3.

**Figure 2:**
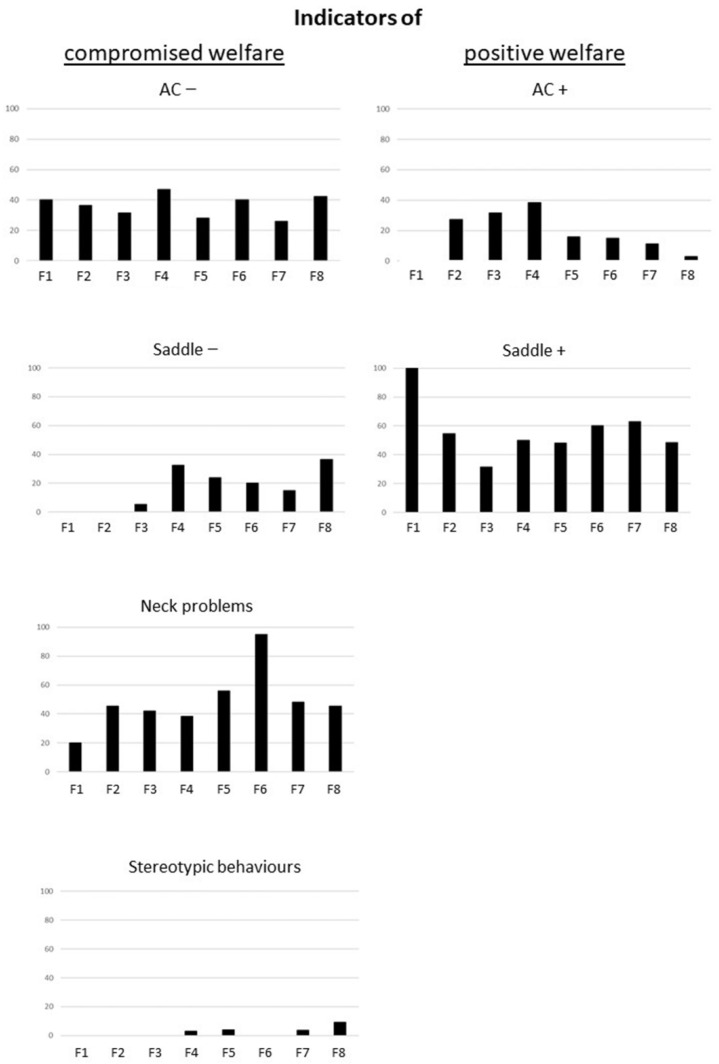
Welfare profiles in the different facilities F1 to F8 (in % of equids per measure, e.g., % of horses with round neck), with, on the left, parameters considered as “positive” in relation to the existing scientific literature, and on the right, parameters considered as “negative” (for more details, see Grandgeorge and collaborators [22]).

There were also clear discrepancies between the facilities in the prevalence of welfare indicators (Figure 2). Thus, for example, stereotypic behaviours were only observed in four facilities, two where the horses were housed indoors (7 and 8) and two where the horses were housed outdoors (4 and 5). In facility 6, almost all horses had a flat or hollow neck, conversely to horses in facility 1, which also all showed positive reactions in the saddle test.

### 3.4. Time Budget (Supplementary Table S1)

**Figure 3:**
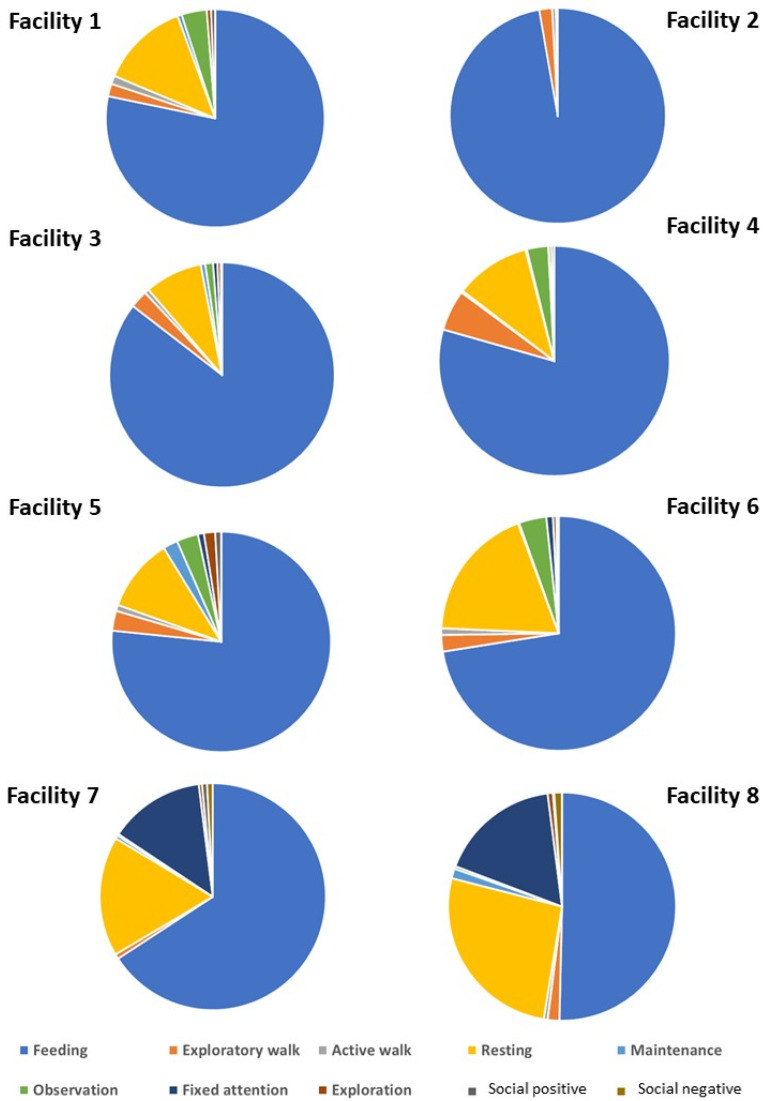
Pie charts showing the distribution of activities in the horses’ time budgets for each facility (mean % of individual time budgets).

Even though feeding was the predominant activity in all facilities, its amount varied according to facility, and this was even more evident for other less frequent activities (Figure 3).

Figure 3 shows that the first four facilities were characterised by a large amount of time spent feeding (especially in facility 2), in exploratory walk and/or in observation behaviours. Facilities 5 and 6 had more diversified profiles, and facilities 7 and 8 had more extreme profiles, characterised by only half the time feeding and the rest mostly standing either resting or in fixed attention.

At the facility level, the time spent in fixed attention was positively correlated with the time spent resting standing and negatively correlated with the time spent feeding (r = 0.86). Indeed, an opposite gradient could be observed from the first four facilities, characterised by more feeding and less fixed attention, to the last three facilities, characterised by more fixed attention and/or resting standing (up to 40% of the time budget). This was not merely due to the context of observation, as the time spent in observation behaviours, which could be performed instead of fixed attention, even indoors, showed an opposite trend.

### 3.5. Time Budget, Management Factors and Welfare

The results are summarised in Table 5.

**Figure 4:**
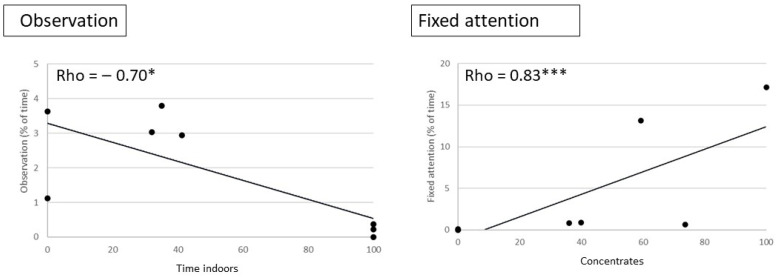
Correlations between the mean type spent (% scans) by horses in the two types of visual attention and management factors in each facility (N = 8 facilities), showing a negative correlation between time spent in observation behaviour and a welfare-challenging factor (time spent indoors) and a positive correlation between fixed attention and the amount of concentrates, considered as a potential risk for the digestive system when in excess. Spearman tests: * *p* < 0.05, *** *p* < 0.001.

**Figure 5:**
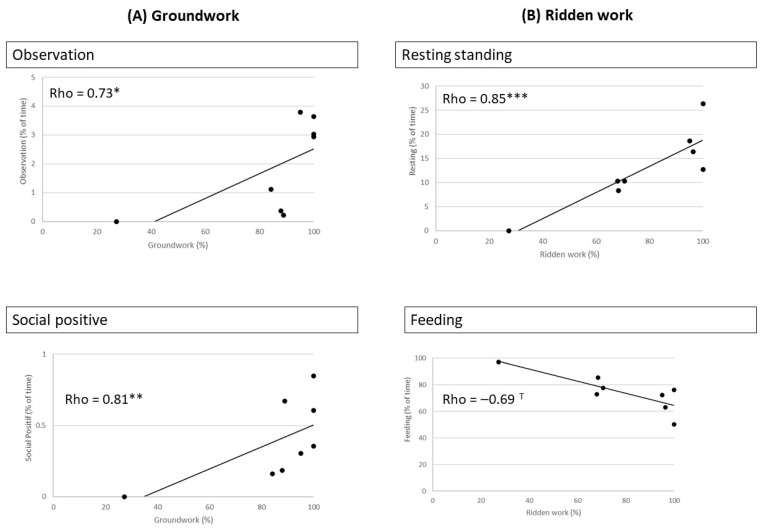
Correlations between the mean time spent by horses in activities (% scans) and the type of working activity in the facility (N = 8 facilities) according to (**A**) groundwork and (**B**) ridden work. Spearman test. T: 0.06 < *p* < 0.05, * *p* < 0.05, ** *p* < 0.01 and *** *p* < 0.001.

There were clear correlations between management factors, including working conditions and the time budgets of the horses at the facility level (Figure 4 and Figure 5):

- Housing conditions: The times spent in observation and active walk were negatively correlated with the median time spent indoors.
- Feeding conditions: The time spent in fixed attention was positively correlated with the % of horses receiving concentrate meals, which also tended to be positively correlated with negative social behaviours (rho = 0.67, *p* = 0.05).
- Working conditions (amount): The time spent in observation and exploratory walk was correlated with the percentage of horses involved in more hours of work (>5.5 h per week).
- Working conditions (type): The times spent in observation behaviours and in positive social interactions were positively correlated with the % of horses doing mostly groundwork. The time spent resting standing was positively correlated with the % of horses doing ridden work, whereas the time spent feeding tended to be negatively correlated with the prevalence of ridden work.

Correlations were also found between welfare measures (from Grandgeorge and colleagues [22] and Figure 2) and time budget measures. The results showed that the more the horses in a facility had positive reactions in the approach–contact test, the more mean time they spent performing exploratory walk, and the more the horses in a facility had their ears mostly forward while foraging, the more mean time they spent feeding and performing exploratory walk and the less time they spent resting standing or in fixed attention.

No other correlation was statistically significant, but the more stereotypic the horses were in a facility, the less time the horses spent feeding (rho = −0.67; *p* = 0.068).

Thus, the best conditions and associated equine welfare (especially facilities 1–4) were characterised by the population of horses that spent more time in calm mobility (85 to 100%, in particular, foraging/grazing, exploratory walk and observation, i.e., scanning of the environment).

## 4. Discussion

This study, based on short observation sessions of horses’ time budgets, confirms the value of such an approach for characterising the «farm atmosphere». Correlations between time budget and welfare data at the individual level have revealed that there are commonalities, even when observations take place in very different contexts and holding conditions. Therefore, this study also shows that such measures allow for comparisons between facilities with very different husbandry systems, where consistent results can be obtained in a limited amount of time spent by the experimenter in the facilities, without disturbing facility routines. Although the indicators involved could differ, the overall valence of activities was confirmed by observations performed indoors and outdoors, at the individual and farm levels. These findings therefore reveal that time budget assessment is a very promising tool for comparing facilities, even when they have very different husbandry systems. One major finding is that the two types of spontaneous visual attention, not even including alarm states, seem to have opposite significance, especially given that most studies have not discriminated between them up to now. Thus, two major issues are raised by the fact that fixed attention, which was also correlated with resting standing, was more evident in facilities where horse welfare was more compromised, whereas observation behaviours, as well as foraging and exploratory walk (all involving some movement either of the body or at least of the ears, neck and head), were more present in facilities where horse welfare was better: (a) a need for re-examining existing ethograms in view of more recent research (e.g., attentional states [46]) and to reach consistent definitions and terminologies that would allow comparisons between studies and (b) the possibility of considering «mobility» on the whole as a sign of better welfare.

On the contrary, resting standing, like fixed attention, was associated with immobility of all or most body parts and seems to be correlated with compromised welfare when occupying a large part of the time budget. Finally, the important differences observed between facilities are all the most remarkable, as the facilities studied were rather attentive to horse welfare, ensuring that most of the horses had roughage ad libitum and at least some time in free turnout. Nevertheless, the data obtained confirm and show how time budgets (i.e., spontaneous behaviours in the home environment) can be drastically affected by management conditions and horses’ welfare states. Thus, whereas some results were not surprising, such as the reduced time in locomotion in some of the facilities where the horses had less access to free turnout, other results are more challenging, such as the differences in the time spent in each of the two types of attention, which did not depend on the housing conditions. Finally, the results obtained confirm the importance of the quality of the working activity for the daily life of animals at other times (i.e., outside work), as differences were found in time budgets according to whether groundwork or ridden work was the predominant activity.

### 4.1. Time Budget as a Tool for On-Farm Welfare Assessment

The present study demonstrates that time budget could be a very promising tool for on-farm welfare assessment in a large variety of domestic situations. The clear correlations between time budget and welfare state at the individual level and the convergent results between observations performed in two very different contexts confirm the value of this approach. While analyses at the individual level allowed more detailed findings, analyses at the facility level confirmed the valence of activities in a more global way, suggesting the possibility of ranking facilities in terms of welfare conditions. Although resource availability could influence time budget, such as food or space resources, the results clearly show that resource availability does not fully explain the differences observed. For example, the time spent in active walk was similar in facility 8, where the horses spent most time indoors, to that observed in some of the facilities where the horses were housed outdoors; the time spent feeding was equally low in the two indoor facilities, although one provided roughage ad libitum (facility 7) and the other provided restricted amounts of roughage (facility 8). A recent study showed that an increased welfare state through particular acoustic stimulations was associated with increased time feeding, while the amount of roughage did not change [51]. The results obtained in this study are especially intriguing, as the facilities studied, all of which practised equine-assisted interventions to some extent, were attentive to their horses’ welfare, with most providing roughage ad libitum and none keeping the horses permanently in single stalls [22]. These findings further demonstrate that even subtle differences in practices may trigger important differences in horse welfare between facilities (e.g., [25]).

Moreover, since the aim was to test a tool for rapid on-farm assessment, the number of observation sessions was small, and the scan sampling led to 33 scans per horse, which may seem like a low number, possibly insufficient to gain exhaustive knowledge of the horses’ time budgets, at least during daytime. Nevertheless, despite this rapid sampling and the low number of facilities (N = 8), the results of the correlations with the management practices, or even the welfare measures, show clear patterns that may be the basis for a reliable assessment on a larger scale.

This study is therefore timely, as on-farm welfare assessment is currently considered as a difficult task, especially in horses, which may live in a large variety of husbandry systems (e.g., [4]). This may be one of the reasons why most studies focus on good practices [52], despite of the fact that, because welfare is subjective, animal-based measures should be the starting point (e.g., [14,53]). Welfare assessment needs to be rapid and based on simple, clearly visible indicators so as to limit risks of under-evaluation [1]. Behaviour is a window into an animal’s internal state, so any change in terms of discomfort, pain or, on the contrary, contentment results in behavioural and/or postural modifications [28]. Reliable behavioural or postural welfare indicators have been identified, but their assessment may require some expertise and specific sampling methods [1,14]. For example, stereotypic behaviours and abnormal repetitive behaviours, which are widely considered to reflect chronic stress (e.g., [35,54]), may be short events or involve long sequences of behaviour. Therefore, scan sampling may lead to under-evaluation, explaining the discrepancy in prevalence found between observational studies performed in similar settings using different sampling methods (e.g., scan sampling: [20,55,56]; all occurrences: [57]). As mentioned by Raspa and colleagues [58], spot sampling may not be appropriate for some behaviours. This is, of course, still more applicable to questionnaire or qualitative assessments (e.g., [59]). The context of observation is also essential, as, for example, stereotypic behaviours may not be expressed, or their type may differ according to the context of observation, for example, during free turnout *versus* in-stall [60]. There have been attempts to apply protocols initially designed for observations in individual stalls to group outdoor housing, but there are difficulties that may further limit the behavioural measures obtained (e.g., AWIN protocol and derivatives [5,6]). We believe that the protocol proposed in our study provides a useful methodology for limiting the biases in most current on-farm assessments.

Thus, our study supports Auer and colleagues’ [15] proposal that time budget could be a useful tool for welfare assessment. Time budget was a favoured approach for describing the behaviour of horses in many of the early studies on feral horses (e.g., [61]), revealing that there were universal trends in all populations studied (e.g., feeding, resting) and that the time budget of animals could vary somewhat according to environmental conditions (e.g., impact of insects on horses’ activities [61]). These trends have also become a reference for domestic conditions, with the idea that the closer the observed time budget is to the feral one, the better (see also [62]). Indeed, time budget is an interesting combination that assesses both resource availability and an animal’s internal state. Thus, Benhajali and colleagues [17] showed that broodmares housed during the day in groups in bare paddocks spent much less time feeding and much more time in active walk than expected from the reference time budget. Providing roughage during daytime in paddocks dramatically changed the time budget, which became closer to the natural reference time budget, but also influenced the quality of other behaviours (e.g., affiliative rather than agonistic social interactions) and the horses’ physiology [39,63]. In these studies, in the absence of access to food resources, the amount of time horses were supposed to feed (60–80% of the time budget) was spent mostly in active locomotion, but also in standing resting or standing alert. Standing immobile also appears to be one major correlate of compromised welfare in the present study, converging with observations performed in anaemic horses that may spend time standing facing a wall [28] and horses experiencing severe pain that spend more time standing and less time feeding [64].

Unfortunately, at present, most studies on time budget in domestic horses are not comparable. Sampling methods can involve scan sampling, but also continuous observations or ad libitum recording, and the ethogram used is not always reported, which would be very important, as, for example, some studies group «standing» (whatever the horse is doing) and «resting» [15]. Similar differences between facilities were also found in the time budget of geriatric horses, which spent more time resting when stabled, but since no difference could be made, for example, between resting standing or resting lying, comparison with our study is limited [65]. The present study clearly contributes to this debate, showing that the time spent in exploratory versus active walk, or standing observing versus standing resting, may not have the same significance when a horse’s internal state is concerned.

### 4.2. Time Budget: The Need for a Revised Consensual Ethogram

As mentioned by Auer and colleagues [15], the lack of homogeneity between studies prevents any possibility of meta-analysis on horse time budgets. Beyond sampling methods, which may not take into account the event versus state types of behaviours [36], one major point is the lack of consistent terminology. Therefore, one main contribution of our study is a proposal for methodological standardisation using a detailed ethogram that clearly differentiates categories, such as exploratory and active walk, or observation and fixed attention or standing resting, avoiding generic terms common in the literature and facilitating comparisons between studies. Indeed, many studies do not provide a defined list of the behaviours observed, while others provide names and definitions of behaviours or activities. Even when the observed items seem similar, they are so different that direct comparison may well be misleading, as shown when considering, for example, activities such as walking or standing immobile (Supplementary Table S1). Although walking is acknowledged as a four-beat gait by all authors, some of the earlier studies separated a «slow walk» from a more rapid and direct walk based on (subjectively assessed) speed and neck posture (e.g., [21]). Other authors included consistency of path or presence or absence of interruptions (e.g., [66]). The combination of low neck posture and interruptions may explain why Joergensen and colleagues [67] chose the term «exploration» for this behaviour. However, this may be misleading, as exploration is used in other studies to describe behaviours that may not require locomotion, such as sniffing the ground or an object [21]. However, we used the term exploratory walk to describe this behaviour, as it seemed to be a consistent and good summary of the above-mentioned descriptions. In contrast, we used «active walk», like Raspa and colleagues [58] and Benhajali and colleagues [17], as a possible standard term to define a more direct, rapid walk with the neck above withers height.

Welfare studies that distinguish between both types of locomotion are scarce but give convergent results. Thus, Benhajali and colleagues [17,39] found that the time spent in active walk «replaced» the time horses would spend eating when mares were in a bare paddock without roughage. Raspa and colleagues [58] mention that active walk is more frequent in inappropriate conditions, whereas in their study, an increase in space led to an increase in slow walk and exploration. In the present study, even in the facilities where the horses were allowed permanent free movement outdoors, we found that the horses did not perform higher gaits, such as trot or canter, despite having the required space, which is in accordance with most studies on feral horses [19]. Exploratory walk was particularly present in the facilities with a higher prevalence of positive welfare, especially those where the human–horse relationship was positive. Thus, often seeing horses in exploratory walk suggests that they may be in a positive welfare state, whereas findings are much more ambiguous for active walk.

The same difficulties apply to behaviours performed while standing immobile. Horses also show a reduced mobility of body parts and a lowered reactivity to stimuli when resting [68], but again, despite a clear definition in Mc Donnell [21], resting standing may also be confused with standing attentive in some studies (Supplementary Table S1). Thus, even in the «classical» early studies, «standing» (alert) and «standing resting» were not always distinguished (e.g., [69], Supplementary Table S1), and Hogan and colleagues [66] mention that they started separating both activities only from 1985 onward. Moreover, our results, in accordance with earlier studies, show that this dichotomic classification is still insufficient to account for the different levels of attention horses can show while standing (e.g., [19,48]).

### 4.3. Visual Attention as a Window into a Horse’s Internal State

Horses, like other mammals, present a gradient from sleep (and its different phases) to resting (also called somnolence or drowsiness), ranging from being in a state of calm attention (i.e., monitoring the environment) to focused attention towards a stimulus or heightened attention when an alarm state is reached (called alarm posture, vigilance, or freezing, according to studies) (e.g., [19,48,70,71]. These different levels of attention can be observed while the horse is standing immobile and may be measured through electrocardiography (ECoG) or electroencephalography (EEG) (e.g., [68,72,73]). There are opposite ways that horses may experience and express their negative perception of their living conditions. On the one hand, horses can exhibit depressed-like states in which they are unresponsive or less responsive to external stimuli. They then stand awake, with their eyes fully open but with an unfocused stare, seemingly oblivious to what happens around them [26,27,74], and, in some cases, even actively «withdraw» from external stimuli by facing a wall for extended periods of time [28]. On the other hand, horses may also become «anxious» and present a high frequency of vigilance/alarm postures or reactivity to novel stimuli (e.g., [75]), which some authors call «hypervigilance» (e.g., [20]). Quantitative EEG measures, performed in the home environment and without any stimulation, have shown that horses in a positive welfare state present an EEG profile typical of calm attention, whereas horses with a compromised welfare state and/or chronic pain present an EEG profile characterised by fast waves, reflecting a chronic heightened alertness [32,33].

Distinguishing these different types of attentional levels therefore appears to be of primary importance. Alarm postures (called here «vigilance», or «freezing» in rodents and other animals (e.g., [76,77]) in horses are characterised by an elevated neck and head posture and constitute an important communicative signal in natural conditions, especially when associated with a raised tail and acoustic signals, such as snores or blows [14,19,47,48]. Group members receive information indicating that there is a potential danger and can get ready for flight (or attack) if needed. Any healthy horse can present this reaction if a frightening stimulus (e.g., an umbrella) appears. Therefore, this behaviour should not be considered as abnormal per se (as in, e.g., [56]), but its potentially high frequency may indicate an anxious state. Thus, young horses show an increase in vigilance postures and the development of stereotypic behaviours shortly after their first single housing in stalls [78]. In the present study, we did not observe any vigilance postures, which may suggest that either none of the horses studied was «anxious» or that the scan sampling method may not be appropriate to detect such brief behaviour. In any case, it would not be an important part of the time budget, as also observed in feral horses, unless a durable danger is present [19].

We found, on the contrary, that horses spent more time in observation behaviour overall and that the time spent in «observation behaviour», defined here as a calm scanning of the environment with mobile ears and slow lateral movements of the neck and head, was much higher in facilities where horse welfare was more positive. This time was positively correlated with the prevalence of groundwork (instead of ridden work) at a facility and negatively correlated with the time spent indoors at the facility level and roughage availability at the individual level. On the contrary, the horses with a compromised human–horse relationship spent less time in observation behaviour, regardless of their main housing (indoors/outdoors). In earlier studies, it has been shown that healthier horses housed in single stalls show more observation behaviours than horses with back problems [44]. In the Principal Component Analysis performed by Gueguen and colleagues [25] on riding school horses, observation was associated with indicators of positive welfare and opposed to stereotypic behaviours, while in the study by Ruet et al. [55], the first axis opposed alert and observation behaviours to stereotypic behaviours, withdrawn postures and aggressiveness, and the second opposed alert and observation behaviours. Regular playback of specially designed sound stimuli was associated with increased welfare states in stall-housed thoroughbreds, which also spent more time in observation behaviours and less time in fixed attention than before and after the playback period [51]. In the present study, the horses in more positive welfare conditions spent little of their time budget in fixed attention, i.e., gazing for more than a second at a stimulus, with their ears and head fixed in the same direction and in total immobility. The neck is then at withers level or slightly above withers, and the tail is not or just slightly raised. This posture, while reflecting focused attention, does not necessarily induce alertness in conspecifics. The time spent in fixed attention went up to 17% of the time in the facility where welfare was most compromised. Moreover, at the individual level, the time spent in fixed attention was correlated with that spent in stereotypic behaviours and was also associated with more concentrate meals and bitted work, two management factors that may have negative consequences (e.g., [22]). Conversely, these two types of attention seem to reflect opposite internal states.

Unfortunately, the lack of distinction between the types of attention and/or clear definitions makes it difficult to determine what type of visual attention is mentioned (Supplementary Table S1). Phelipon and colleagues [56] use watching or scanning, on the one hand, and alert behaviour (the definition of which seems to correspond to our vigilance postures), on the other hand, but they do not mention a fixed attention that would neither be observation nor vigilance, suggesting that it was included in one of these two categories. The same applies to «standing alert» and «standing relaxed» in Raspa and colleagues’ study [58], which may represent either vigilance, fixed attention and resting or observation, or also vigilance + fixed attention + observation and resting. Glauser and colleagues [79] use the term «inactive stance» versus «active stance», which may or may not correspond to our categories, or may include depressed-like (withdrawn, apathetic) states where horses stand immobile with a high fixity of the whole body and an unfocused stare, with eyes fully open (e.g., [27,80] (not observed in our study)). Interestingly, there seems to be a gradient in the «fixity» of horses’ behaviours while standing, from observation behaviours, mostly associated with positive welfare indicators, to fixed attention, apparently more associated with negative indicators, and vigilance, associated with alarm or anxiety; at the extreme, depressed-like horses even show a reduced rate of blinking and a low reactivity to external stimuli [27,74]. In monkeys, socially tolerant groups are characterised by more monitoring behaviours (i.e., mobile visual attention), whereas prolonged gazes towards the dominant individuals characterise «despotic» social systems; these two systems lead to very different internal states for the group members [41].

### 4.4. Mobility as a Sign of Positive Welfare

Overall, we found that the time budget of the horses living in the two facilities where horse welfare was the most compromised was indeed characterised by a lack of mobility, whether locomotory per se or while feeding on roughage (only half of the time) or observing with head, ears and neck movements: they could spend almost half of the time standing either in fixed attention or resting. Camargue horses increase their time standing when space is reduced [61], and meat horses increase their time standing when the stocking density is high [58,81]. Some studies mention that standing, whether resting or alert, is the most (or one of the most) represented activity in stall-housed horses [48] (65%, [82]), a type of housing that compromises welfare (e.g., [14,80,83]). Standing also increases when conditions become harsh, like winter in extreme climatic conditions [16,67,84]. In the domestic situation, Price and colleagues [85] found an increase in the time spent standing in horses that underwent surgery, and Elia and colleagues [86] found an increase in standing when horses were given more concentrates, which was also observed here with fixed attention.

In the present study, as mentioned above, a long time spent standing immobile, whether resting or in fixed attention, seems to be indicative of a rather negative state. Overall, these observations of standing immobile, whether in fixed attention or resting, show the ambiguous status of some behaviours, such as resting, which may arise from relaxation but also from “boredom”, often reported as “inactivity” overall in animals [87].

Conversely, exploratory walk, observation behaviours and feeding (as well as positive social interactions) were most represented in the facilities where management conditions and horse welfare were best and could be positively correlated with positive welfare indicators, such as a positive human–horse relationship, and negatively with restricted life conditions (indoor housing, more concentrates). These findings may help describe how a satisfied, satiated [6] or content [1] horse behaves; thus, they may establish time budget assessment as a basis for describing positive welfare in horses, such as a «calm of mind» or calm attentional state [14,52]. They also align with findings that snort production, an acoustic signal generally observed in calm positive situations [38,83,88], was correlated with time in observation behaviours and feeding when horses were observed (and living) outdoors.

Finally, at the facility level, both the times spent in observation and positive social interactions were correlated with the prevalence of groundwork in the facility, whereas the prevalence of ridden work time was positively correlated with the time the horses spent in standing resting and negatively correlated with feeding. At the individual level, the time spent standing resting or in fixed attention was higher in the horses that worked bitted, whatever the context of the time budget assessment. Inappropriate bit actions are frequent in riding schools and are more prone to happen in equine-assisted activities [22,29,89]. Earlier analyses had shown that ridden work was associated with lower welfare than groundwork [22], confirming that the type of activity may influence welfare [57] but also the type of attention that horses may spontaneously develop when exposed to visual stimuli [45]. The finding that the prevalence of horses with more than 5.5 h work per week was correlated with the time spent in observation and exploratory walk may further confirm that work quality, more than work quantity, may influence a horse’s welfare state. In the present case, groundwork and unbitted work were well represented in the facilities that offered equine-assisted interventions and may have been appreciated by the horses. Further studies on a larger and more diversified sample of facilities and using the same methodology are now needed to refine the protocol, further disentangling the respective influences of welfare state, context of observation and resource availability on the observed time budget and confirming the use of key activities for this rapid assessment. In future studies, it would also be interesting to test different durations of observation so as to determine what actual threshold is reliable enough, a question that remains underscored in all existing on-farm welfare assessments [1].

## 5. Conclusions

This study confirms the interest and usefulness of time budgets for on-farm welfare assessment, allowing a quick and reliable overview of horse welfare states in a facility. More precisely, the results indicate that a predominance of fixed attention over observation behaviours, especially if associated with a significant (more than 15%) time spent resting standing, may be indicative of compromised welfare and should prompt an investigation of the factors involved. Conversely, when horses spend most of their time in calm mobility (scanning the environment, exploratory walk, feeding), it is likely that welfare conditions in the facility are positive. Further studies on a larger and more diversified sample of facilities using the same methodology are now needed to refine the protocol, further disentangling the respective influences of welfare state, context of observation and resource availability on the observed time budget and confirming the choice of key activities for this rapid assessment. Overall, this study makes a significant contribution to the methodology and nomenclature concerning the use of time budgets for welfare assessment and highlights new approaches to the assessment of positive welfare.

## Figures and Tables

**Table 1:** Characteristics of the equids and general management practices in the facilities (a few individual horses may differ in each facility) (modified from Grandgeorge and colleagues [22]). NA corresponds to “not applicable”. The context of time budget observations corresponds to the main housing context. Mean +/−SD = mean value and standard deviation based on the individual data from the facility.

Facility Number	Equid Number	Mean Age (yo ± SE)	Outdoor Housing (In Group)		Indoor Housing				Roughage (Hay/Grass)	Total Amount of Pellets per Day (Litres)	Work per Week (Hours)
			Outdoor Housing per Day (Hours)	Type	Hours per Day	Single or Group	Type	Bedding	Total Amount per Day		
1	5	10 ± 6	24	Pasture	0	NA	NA	NA	Ad libitum	0	3 ± 1.9
2	11	16 ± 6	12	Pasture	12	Group	Collective stall	Straw	Ad libitum	0	1.25 ± 0.5
3	20	14 ± 6	24	Pasture	0	NA	NA	NA	Ad libitum	0.25	4.4 ± 1.6
4	34	15 ± 5	22 ± 2	Pasture	1 ± 2	Both	Single- or collective stall	Straw	Ad libitum	0	11 ± 3
5	25	11 ± 3	17 ± 9	Paddock	7 ± 10	Single	Single-stall	Straw	Ad libitum	4	10 ± 2.8
6	19	14 ± 6	21 ± 5	Pasture	3 ± 5	Single	Single-stall	Sawdust	Ad libitum	0.5	8.6 ± 2.3
7	27	13 ± 8	1 ± 0.3	Paddock	23 ± 0.7	Both	Single- or collective stall	Straw	Ad libitum	3.7	3.2 ± 2.3
8	33	15 ± 5	0.8 (1 day of 6 h once a week)	Pasture or paddock	24 (6 days a week) + 18 h once a week	Both	Single- or collective stall	Wood shavings	2 to 6 kgs	0.5	<5.5 h per horse

**Table 2:** Definitions of the activities recorded during the time budget observation scan sampling sessions. Based on Waring [19], Mc Donnell [21] and indicated references when needed.

Activity Categories	Definitions
Feeding	Grazing (mostly) at the grass level and mostly mobile with one or two steps between successive grass intakes or eating and ingesting grass, bushes, leaves, and roughage, which is mostly observed with the horse standing immobile, neck at resource height.
Locomotion	Continuous movement of the horse’s legs and neck above ground, without any food intake or food chewing. 1. Exploratory walking (or slow walk): Movement forward in the slowest (four-beat) of the mammalian quadrupedal gaits. Neck low and no clear direction. 2. Active walk: Movement forward in the slowest (four-beat) of the mammalian quadrupedal gaits. Neck higher, looking ahead, with clear direction of movement. 3. Trot, canter (not observed during sessions): Three- and 4-beat gaits.
Maintenance	Self-scratching, scratching, rubbing against an object, rolling, or yawning.
Exploration	Sniffing, licking, or biting (without ingestion) items in the environment.
Social interactions	1. Positive (=ears forward): Approaching, sniffing, and mutual grooming. 2. Negative (=ears backwards): Approaching, threatening to bite, biting, threatening to kick, and kicking.
Resting	Standing immobile on 3 or 4 legs (no resting lying observed) with neck horizontal or slightly elevated, ears and neck mobile, and slowly with ears in variable positions, and eyes partially closed.
Visual attention	1. Observation (or monitoring behaviour): Standing with neck horizontal or slightly above horizontal and scanning the environment by moving the head laterally, or occasionally gazing for a short moment at environmental stimulus [25,44]. 2. Fixed attention (or prolonged gazing): Standing immobile with ears and head fixed in the direction of gazes, neck horizontal or slightly above horizontal [25,46]. 3. Vigilance (not observed during sessions): Alarm posture with fixed ears and neck, very high neck posture, and tail raised, can be associated with snores or blows (e.g., [47,48]).

**Table 3:** Significant correlations (N = 174 equids) (continuous quantitative data: Spearman tests), comparisons (categorical data: Kruskal–Wallis or Mann–Whitney tests) (*p* < 0.05) and tendencies (0.05 > *p* < 0.08 in italic) between time budget (% scans of activities) and welfare measures and between time budget and management factors. Blue colour indicates positive correlations and comparisons; brown colour indicates negative correlations and comparisons.

Time Budget	Welfare Indicators +	Welfare Indicators −	Management Factors +	Management Factors −
Feeding (indoors)	Ears forward (rs = 0.366, *p* = 0.02, U = 167.5, *p* = 0.003) Positive at saddle test (U = 310.5, *p* = 0.04)	Ears backwards (rs = −0.413, *p* = 0.001) Stereotypies (rs = −0.552, *p* = 0.02)		Restricted roughage (U = 615, *p* = 0.011) 2–3 meals of concentrates (KW = 9.08, *p* = 0.01) Working bitted (U = 661, *p* < 0.001)
Feeding (outdoors)	*Positive at saddle test * *(U = 1287.5, p = 0.055)*		Roughage ad libitum (U = 49.5, *p* < 0.001) Not ridden (U = 915, *p* < 0.001)	
Maintenance (outdoors)	Round neck (U = 1191, *p* = 0.005)			
Social positive (indoors)	Round neck (U = 530.5, *p* = 0.029)			
Social positive (outdoors)	Round neck (U = 1362, *p* = 0.01)			
Social negative (indoors)				Three or + meals of concentrates (KW = 8.74, *p* = 0.013)
Social negative (outdoors)				
Exploratory walk (indoors)	*Positive reactions at saddle test * *(U = 342.5, p = 0.057)*			
Exploratory walk (outdoors)	Ears forward (rs = −0.172, *p* = 0.05)			
Active walk (indoors)				Restricted access to roughage (U = 364.5, *p* = 0.02)
Active walk (outdoors)				No groundwork (U = 1647.5, *p* = 0.037)
Resting standing (indoors)	Ears forward (rs = −0.287, *p* = 0.026) Positive reactions at saddle test (U = 586.5, *p* = 0.035)	Ears backwards (rs = 0.262, *p* = 0.04)		Working bitted (U = 280.5, *p* = 0.045)
Resting standing (outdoors)	Ears forward (rs = −0.272, *p* = 0.003)	Ears backwards (rs = 0.222, *p * = 0.018)		*One or more meals of concentrates * *(KW = 1041.5, p = 0.056)* More than 3 h work per week (KW = 11.43, *p* = 0.0009)
Observation (indoors)		*Negative reactions at AC test (U = 472.5* , *p = 0.06)*		
Observation (outdoors)		Negative reactions at AC test (U = 1206, *p* = 0.03)	Roughage ad libitum (U = 737, *p* = 0.037)	
Fixed attention (indoors)		Ears backwards (U = 519, *p* = 0.0018) Stereotypic behaviours (rs = 0.292, *p* = 0.02)		Three or + meals of concentrates (KW = 8.55, *p* = 0.014) Working bitted (U = 237.5, *p* = 0.007)
Fixed attention (outdoors)				Three or + meals of concentrates (KW = 1086.5, *p* = 0.035) *Working bitted * *(U = 1083, p = 0.059)*

**Table 4:** Comparison of management practices between the facilities using a v-test (from Grandgeorge and collaborators [22]). The v-test facilitates the description of the classes of a partition (e.g., after an automatic classification). Test values are calculated for each continuous variable or category of a qualitative variable. They are measurements of the distance between the within-class value and the overall value. The results indicate the categories that have a v-test higher than the value on one of the dimensions tested. Only the statistically significant differences are indicated. When there is no value, this means that the facility is average compared to the others. As an example, facilities 1, 3, 4, 5, and 6 offered more time outdoors than expected from the average of the 8 facilities, and facilities 2, 7 and 8 offered less; facilities 1, 2, 4 fed their horses less concentrates, facilities 3 and 8 fed more, and facilities 5, 6 and 7 fed an average proportion. Values above average are indicated in bold; values under average are in light.

			Facility 1	Facility 2	Facility 3	Facility 4	Facility 5	Facility 6	Facility 7	Facility 8
**Management**	Outdoor housing	**v-test**	**2.63**	−2.94	**5.35**	**2.14**	**2.78**	**2.15**	−4.85	−5.47
		*p*-value	0.009	0.003	8.64 × 10^−8^	0.033	0.006	0.031	1.24 × 10^−6^	4.41 × 10^−8^
	Ad. Lib. Roughage	**v-test**								−10.94
		*p*-value								7.63 × 10^−28^
	Concentrates	**v-test**	−2.09	−3.15	**2.56**	−5.98				**6.90**
		*p*-value	0.037	0.002	0.010	2.24 × 10^−9^				5.29 × 10^−12^
	>5.5 h/d work	**v-test**		−2.72		**7.11**	**5.24**		−2.81	−5.50
		*p*-value		0.007		1.19 × 10^−12^	1.57 × 10^−7^		0.005	3.83 × 10^−8^
	Bit	**v-test**	−3.51	−5.31	−3.42	**3.75**	**3.47**	**2.53**		
		*p*-value	4.42 × 10^−4^	1.12 × 10^−7^	0.001	1.73 × 10^−4^	0.001	0.011		
	Groundwork	**v-test**		−4.39			**3.13**		**2.36**	**3.25**
		*p*-value		1.14 × 10^−5^			0.002		0.018	0.001
	Ridden work	**v-test**		−6.77	**2.27**					
		*p*-value		1.28 × 10^−11^	0.023					

**Table 5:** Significant correlations (*p* < 0.05) and tendencies (0.05 > *p* < 0.08 in italics) between time budget and welfare measures and between time budget and management at the facility level. Green colour indicates positive correlation; red colour indicates negative correlation.

Time Budget	Welfare Indicators +	Welfare Indicators −	Management Factors +	Management Factors −
Feeding	*Ears Forward (rho = 0.84, p = 0.009)*			*% horses ridden (rho = −0.69, p = 0.056)*
Social positive			*% horses doing groundwork (rho = 0.81, p = 0.016)*	
Exploratory walk	*Positive reactions at AC test (rho = 0.79, p = 0.028)* *Ears forward (rho = 0.84, p = 0.009)*			*>5.5 h/wk working (rho = 0.72, p = 0.045)*
Exploration			*% horses doing groundwork (rho = 0.90, p = 0.002)*	
Active walk				*Median time spent indoors (rho = −0.75, p = 0.037)*
Resting standing	*Ears forward (rho = −0.83, p = 0.011)*			*% horses doing ridden work (rho = 0.85, p = 0.007)*
Observation			*% horses doing groundwork (rho = 0.73, p = 0.035)*	*Median time spent indoors (rho = −0.70, p = 0.05)* *>5.5 h/wk working (rho = 0.72, p = 0.045)*
Fixed attention	*Ears forward (rho = −0.73, p = 0.025)*			*% horses fed concentrates (rho = 0.83, p = 0.01)*

## Data Availability

The raw data supporting the conclusions of this article will be made available by the authors on request.

## Supplementary Materials

The following supporting information can be downloaded at https://www.mdpi.com/article/10.3390/ani16101557/s1: Table S1: Examples of terminologies used in different studies for standing activities and walking. Ø corresponds to cases where there does not seem to be an equivalent to our categories; ? corresponds to cases where we are not sure whether this is equivalent. Additional references in the cells indicate when authors used the same terminology. References [11,12,19,21,53,54,56,59,64,65,66,79,81,90,91,92,93,94,95] are cited in the Supplementary Materials; Table S2: Time budget (% time of activities) at the facility level (mean of individual TBs per facility) (N = 8 facilities).
