# Supplementary material for: Farm Atmosphere: Calm Attention and Mobility Characterise Positive Horse Welfare
Source: Animals (Basel). 2026 May 20;16(10):1557. doi: 10.3390/ani16101557 (PMC13203647; doi:10.3390/ani16101557)
Supplement: Supplementary file 1 [file animals-16-01557-s001.zip › SuppTable1.pdf]

	Domestic/feral horses													Other equids
	Our team	***Feral/free-roaming***			***Domestic/welfare***						***Przewalski***			***Zebra***
	This study	Keiper (1985)<br>Mc Donnell (2003)<br>Tyler (1972)	Duncan (1979, 1980, 1985)	Feist (1971) Waring (1983, 2003)	Ruet et al. (2024)	Phelipon et al. (2024)	Raspa et al. (2020 a,b)	Glauser et al. (2015)	Joergensen & Boe 2007	Annan et al. 2023(Duncan & Vigne, 1979)	Hogan et al. 1988	Souris et al. 2007	Ruckebusch 1972	Schilder and Boer 1987
**STANDING**	**Resting**<br><br>Standing immobile with ears in variable positions, eyes partially closed	**Rest Standing**<br>inactive in a relaxed posture, usually with head slightly lowered, eyes partly or nearly closed, and often bearing weight on three legs (one hind leg slightly flexed). With deeper drowsiness (transition between wakefulness and sleep), the lips relax and the ears rotate laterally.	**Standing resting**<br>Standing immobile, usually with one hind leg relaxed, eyes partially or wholly closed, head held low, ears pointing in different directions. Ears and tail may move				**Resting standing**<br>Standing with expression relaxed	*Inactive stance?*	**Standing**<br>Horse stands inactive with 4 feet or 1 hooked up in a resting position, relaxed ears and head, eyes open	**Resting standing**	*Grouped with standing*	**Resting**<br>Standing, relaxed, neck horizontal or low, head relaxed, nose towards ground, eyes closed or half shut, ears in lateral position or directed behind, one leg flexed	**Drowsiness**<br><br>Or somnolence<br><br>Low and high EcoG waves Little awareness of environment	
	**Observation** *or monitoring behaviour*<br><br>Standing immobile, with neck horizontal or	**Ø**	**Ø**		**Observation posture**<br>The horse is standing in the individual box, its neck is carried horizontally or	**Watching behavior**<br>Standing still with the neck held horizontally or slightly raised, while the ears and neck	***Standing relaxed?***	**Inactive stance**	***Included in standing?***	**Interested**<br>Interested in surroundings, watching people or other horses, alert but calm	***Grouped with standing?***		***Alert wakefulness?***	

	slightly elevated, ears and neck mobile, and slowly scanning the environment by moving the head laterally or occasionally gazing for a short moment at environmental stimuli<br><br>Used also by Gueguen et al. (2025), Rochais et al. (2016)				mid-high in relation to the line of the back and it moves slowly from left to right or up and down. Its ears are mobile, its eyes are open and almond-shaped or slightly round, and its nostrils are long/not dilated. Its tail is relaxed along the body. The horse can stand still for one or two seconds before changing its neck position	remain mobile. The horse slowly surveys its surroundings by moving its head side to side or briefly looking at environmental stimuli occasionally								
	**Fixed attention**<br><br>Ears and head fixed in the direction of gazes, neck horizontal or slightly above horizontal<br><br>Used also by Gueguen et al. (2025), Rochais et al. (2017)	**Stand alert** Rigid stance with the neck elevated and the head oriented toward the object or animal of focus. The ears are held stiffly upright and forward, and the nostrils may be slightly dilated.<br><br>Used also by Tyler (1972)	**Standing alert** Standing with head raised or moving, ears oriented in the same direction and eyes fully open. *Drinking included*	**Stare/stand stare**	***With alert posture ?***	***With alert behaviour?***	**Standing alert/ Watching** Horse standing attentive observing surrounding	**Active stance**	**Standing alert** Horse stands with ears and eyes focused	**Standing observing** Standing while being alert, usually at door or window	**Standing** No movement, standing squarely on all feet, head erect, ears forward, attention focused on a distant object or sound	**Standing** Standing with sustained position, head high, neck held with tension, ears forward *or moving to intercept surrounding sounds*	**Alert wakefulness**	**Attention face**

	**Vigilance**<br><br>Alarm posture with fixed ears and neck, very high neck posture, tail raised, can be associated with snores or blows<br><br>Used also by Berger (1986), Kiley-Worthington (1976), Wolff et al. (1997)	**Stand alert**<br>*Alarm responses* — highly stylized responses and postures to signs of danger that serve to alert the group. The responses typically provide auditory, olfactory, and/or visual signals to the group. Typical alarm signals in the horse are the alert stance with eyes and ears pointing toward the threat and the blow or snort vocalization		**Alarm**<br>stronger form of the alert stance, with eyes widely open and sclera showing. The behavior may include an arched neck and flared nostrils	**Alert posture**<br>The horse stands still, its neck is carried mid-high to high in relation to the line of the back, its ears are fixed, its eyes are open and round and the sclera is sometimes visible, and its nostrils are round/dilated. The horse appears muscularly tense. Its tail is slightly raised. The horse may also express a snort or a blow	**Alert behaviour**<br>=<br>*hypervigilance (abnormal behaviour)*<br>Remaining vigilant in position, with awareness and a raised neck, carefully surveying the environment, occasionally shifting either head or ears<br><br>also used by Young (2012)	**Alarmed**		**Standing alert**<br>Horse stands with ears and eyes focused	**Standing**<br>No movement, standing squarely on all feet, head erect, ears forward, attention focused on a distant object or sound	
**WALKING**	**Exploratory walk**	**Slow walk**<br>Movement forward in the slowest (four beat) of the mammalian quadrupedal gaits.<br>Neck low	**Milling**				**Slow walk**	**Walking with head low**	**Exploration**	**Milling**<br>Walking bouts (<3 sec) interrupted by steps, pauses and numerous direction changes, no consistent path followed	

	**Active walk**	**Alert walk** Movement forward in the slowest (four beat) of the mammalian quadrupedal gaits. Neck higher, looking ahead	Locomotion/walk	**Active walk**	**Walking with head high**	**Walk**		

<u>References</u>

Annan, R., Trigg, L. E., Hockenhull, J., Allen, K., Butler, D., Valenchon, M., & Mulla, S. (2023). Racehorse welfare across a training season. *Frontiers Veterinary Sciences*, *10:1208744*.

Duncan, P. (1985). Time-budgets of Camargue horses III. Environmental influences. *Behaviour*, *92*(I-2), 188-208.

Duncan, P., & Cowtan, P. (1980). An unusual choice of habitat helps Camargue horses to avoid blood-sucking horse-flies. *Biology of behaviour*, *5*, 55-60.

Duncan, P., & Vigne, N. (1979). Effect of group-size in horses on the rate of attacks by blood-sucking flies. *Animal Behviour*, *27*(2).

Feist, & J.D. (1971). *Behavior of feral horses in the Pryor Mountain Wild Horse Range*.

Glauser, A., Burger, D., van Dorland, H. A., Gygax, L., Bachmann, I., Howald, M., & Bruckmaier, R. M. (2015). No increased stress response in horses on small and electrically fenced paddocks. *Applied Animal Behaviour Science*, *167*, 27-34.

Hogan, E.S., Houpt, K. A., & Sweeney, K. (1988). The effect of enclosure size on social interactions and daily activity patterns of the captive Asiatic wild horse (E*quus przewalskii)*. *Applied Animal Behaviour Science*, *21*, 147-168.

Jørgensen, G. H. M., & Bøe, K. E. (2007). Individual paddocks versus social enclosure for horses. In *Horse behaviour and welfare*. Wageningen Academic.

Keiper, R. R. (1985). *The assateague ponies*. Tidewater Publishers.

McDonnell, S. (2003). *A practical field guide to horse behavior: the equid ethogram*. The Blood-Horse Inc.

Phelipon, R., Hennes, N., Ruet, A., Bret-Morel, A., Górecka-Bruzda, A., & Lansade, L. (2024). Forage, freedom of movement, and social interactions remain essential fundamentals for the welfare of high-level sport horses. *Front Vet Sci*, *11*, 1504116. https://doi.org/10.3389/fvets.2024.1504116

Raspa, F., Tarantola, M., Bergero, D., Bellino, C., Mastrazzo, C. M., Visconti, A.,…Valle, E. (2020). Stocking Density Affects Welfare Indicators in Horses Reared for Meat Production. *Animals (Basel)*, *10*(6). https://doi.org/10.3390/ani10061103

Raspa, F., Tarantola, M., Bergero, D., Nery, J., Visconti, A., Mastrazzo, C. M.,…Valle, E. (2020). Time-Budget of Horses Reared for Meat Production: Influence of Stocking Density on Behavioural Activities and Subsequent Welfare. *Animals (Basel)*, *10*(8). https://doi.org/10.3390/ani10081334

Ruckebusch, & Y. (1972). The relevance of drowsiness in the circadian cycle of farm animals. *Animal Behaviour*, *20*, 637-643.

Ruet, A., Lemarchand, J., Briant, C., Arnould, C., & Lansade, L. (2024). A field approach to observing changes in behavioural welfare indicators over 2 years in stabled horses. *Animal*, *18*(4), 101120. https://doi.org/10.1016/j.animal.2024.101120

Schilder, M.B.H., & Boer, P. L. (1987). Ethological investigations on a herd of Plains Zebra in a safari park: Timebudgets, reproduction and food competition. *Applied Animal Behaviour Science*, *18*, 45-56.

Souris, A. C., Kaczensky, P., Julliard, R., & Walzer, C. (2007). Time budget, behavioral synchrony and body score development of a newly released Przewalski's horse group E*quus ferus przewalskii*, in the Great Gobi B strictly protected area in SW Mongolia. . *Applied Animal Behaviour Science*, *107*, 307-321.

Tyler, & S.J. (1972). The behavior and social organization of the New Forest ponies. *Animal Behaviour Monographies*, *5*.

Waring, G. H. (2003). *Horse behavior* (2nd edition ed.). Noyes Publications, William Andrew Publishing.
