# Supplementary material for: Farm Atmosphere: Calm Attention and Mobility Characterise Positive Horse Welfare
Source: Animals (Basel). 2026 May 20;16(10):1557. doi: 10.3390/ani16101557 (PMC13203647; doi:10.3390/ani16101557)
Supplement: Supplementary file 1 [file animals-16-01557-s001.zip › SuppTable2.pdf]

Facility number	Feeding	Exploratory walk	Active walk	Resting	Maintenance	Observation	Fixed attention	Exploration	Social Positive	Social Negative
1	76.4	1.8	1.2	12.7	0.6	3.6	0.0	0.6	0.6	0.0
2	97.2	1.9	0.6	0.0	0.3	0.0	0.0	0.0	0.0	0.0
3	85.5	2.6	0.6	8.3	0.6	1.1	0.6	0.5	0.2	0.0
4	77.7	5.6	0.3	10.3	0.2	2.9	0.1	0.4	0.4	0.0
5	73.1	2.8	0.8	10.3	2.1	3.0	0.8	1.6	0.8	0.0
6	72.3	2.3	0.9	18.6	0.2	3.8	0.9	0.5	0.3	0.0
7	63.1	0.7	0.0	16.4	0.4	0.2	13.1	0.4	0.7	0.8
8	50.2	1.7	0.6	26.4	1.4	0.4	17.2	0.7	0.2	1.1
